# Promising Endophytic *Alternaria alternata* from Leaves of *Ziziphus spina-christi*: Phytochemical Analyses, Antimicrobial and Antioxidant Activities

**DOI:** 10.1007/s12010-022-03959-9

**Published:** 2022-05-17

**Authors:** Rasha Y. Abd Elghaffar, Basma H. Amin, Amr H. Hashem, Amira E. Sehim

**Affiliations:** 1 Botany and Microbiology Department, Faculty of Science, Benha University, Benha, Egypt; 2 The regional Center for Mycology and Biotechnology, Al-Azhar University, Cairo, 11787 Egypt; 3 Botany and Microbiology Department, Faculty of Science, Al-Azhar University, Cairo, 11884 Egypt

**Keywords:** Fungal endophytes, Antimicrobial activity, Antioxidant activity, Cytotoxicity, Phytochemical screening

## Abstract

Fungal endophytes are considered one of the most important reservoirs of bioactive compounds which defeat resistant microbes. In our study, endophytic *Alternaria alternata* was isolated from *Ziziphus spina-christi* and identified morphologically and genetically with accession number OM 331,682. Preliminary phytochemical screening of ethyl acetate (EA) crude extract of *A. alternata* revealed that this extract contains alkaloids, tannins, flavonoids, glycosides, phenols, and terpenoids. Moreover, the extract was analyzed using gas chromatography-mass spectrometry (GC–MS) which verified the presence of numerous bioactive compounds. Antimicrobial results illustrated that EA crude extract exhibited promising antimicrobial activity against Gram-negative bacteria (*Escherichia coli* ATCC 11229, *Proteus vulgaris* RCMB 004, *Pseudomonas aeruginosa* ATCC 27853, and *Klebsiella pneumonia* RCMB 003), Gram-positive bacteria (*Bacillus subtilis* RCMB 015, *Staphylococcus aureus* ATCC 25923, and *Staphylococcus epidermidis* ATCC 14990), and unicellular fungi (*Candida albicans* ATCC 90028). Ultrastructure study of treated *K. pneumonia* showed remarkably elucidated destruction of the cell wall and cell membrane and leakage of cytoplasmic materials. Furthermore, the extract has potential antioxidant activity where IC_50_ was 409 µg/mL. Moreover, this extract did not show any toxicity on Vero normal cell line. These findings confirmed that the endophytic *A. alternata* from *Z. spina-christi* is a promising source of bioactive compounds which can be used in different biological applications.

## Introduction

The emergence of pathogenic bacteria and fungi resistant to commercial drugs is a relevant problem faced by health services; this is due to the microbes acquiring new mechanisms to resist antimicrobial agents [1, 2]. Therefore, the discovery of effective antimicrobial agents is required. Fungal endophytes can live in plant tissues without producing any apparent symptoms or obvious harm effects to their hosts [3]. They have been existing in all plant species studied [4], colonizing the area underneath the epidermal tissue, absorbing their food from the plants, and improving plant growth of host and protecting it from pathogens by inhibiting the growth of plant pathogen and inducing the systematic resistance of plant as defense mechanisms [5]. Fungal endophytes are considered one of important reservoirs of bioactive compounds which have different biological activities such as antimicrobial, antioxidant, anticancer, antiviral, and antimalarial activities [3, 6, 7]. These activities are attributed to different effective secondary metabolites such as alkaloids, phenols, steroids, terpenoids, saponins, glycosides, tannins, and flavonoids [8, 9]. Endophytic *Alternaria* spp. have variety of biological activities, such as antimicrobial, antioxidant, antiviral, anticancer, and phytotoxic activities [10, 11]. Recently, more metabolites with different bioactivities from *Alternaria* fungi have been extracted and structurally characterized. Therefore, exploring of fungal endophytes which live in medicinal plant enables us to discover new metabolites [12]. Fungal endophytes reside in medicinal plant which grows in natural habitat. Stems, leaves, and roots are a huge reservoir for these endophytic fungi which can be used in different biological application as cytotoxic, antibacterial, antifungal, antiviral, and antioxidant activities [13]. *Ziziphus* also known as “Sedra”s is an important genus of the family Rhamnaceae found growing extensively in arid and semi-arid regions and represented byy135–170 species [14]. It was reported that the fungal crude extract of *Trichoderma viride* isolated from the medicinal plant of *Ziziphus mauritiana* was displayed anticancer activity against HeLa cell line [15]. Leaf extract of *Z. mauritiana* has potential antimicrobial against *B. cereus*, *S. aureus*, *S. pneumoniae*, *B. subtilis*, *P. vulgaris*, *E. coli*, and *C. albicans* [16]. Although *Ziziphus spina-christi* (*Z. spina-christi*) is common in the environment, few studies studied their associated endophytic fungi [17]. Herein, this study is conducted to isolate fungal endophytes from medicinal plant *Z. spina-christi* and evaluate their antimicrobial, antioxidant, as well as cytotoxicity activities.

## Materials and Methods

### Collection of Plant Materials

Disease free and mature leaves of *Z. spina-christi* were collected from Menofia Governorate. Fresh leaf samples were transported to the lab under aseptic conditions for further isolation of endophytic fungi.

### Test Microorganisms

Pathogenic microorganisms used in this study were kindly provided from the culture collection unit at the Regional Center for Mycology and Biotechnology (RCMB), Al-Azhar University against Gram-negative bacteria (*E. coli* ATCC 11229, *P. vulgaris* RCMB 004, *P. aeruginosa*, and *K. pneumonia* RCMB 003), Gram-positive bacteria (*B. subtilis* RCMB 015, *S. aureus* ATCC 25923, and *S. epidermidis* ATCC 14990), and unicellular fungi (*C. albicans* ATCC 90028.

### Isolation of Endophytic Fungi

Isolation of endophytic fungi was performed after removing epiphytes from leaf surface with water according to the method described by Strobel and Daisy [18] with slight modification. Firstly, fresh leaves were washed thoroughly in running tap water for 10 min and sterilized in series with 70% ethanol forr1 min and 1.0% sodium hypochlorite (NaOCl) (v/v) for 1 min and further cleaned by passing through two sets of sterile distilled water. After sterilization, leaves were cut into small pieces, 1 cm long, and placed on a plate containing potato dextrose agar (PDA) medium amended with 250 µg/mL streptomycin to suppress bacterial contamination. The plates were incubated at 28 °C until the fungal mycelial started growing on the samples [19, 20]. The last wash water was spread onto PDA plates and served as a negative control to evaluate the success of sterilization [21]. Hyphal tips of emerging colonies from the cultivated leaf sections were sub-cultured on fresh PDA plates to get its pure culture [22].

### Morphological and Molecular Identification of Fungal Endophytes

The isolated fungal endophyte was identified morphologically according to Khalil et al. [6]. Macroscopic morphological features including color, texture, and diameter of colonies and microscopic characteristics including vegetative and reproductive structures of the fungus were observed [23–28]. The genomic DNA was isolated and purified using Quick-DNA Fungal Microprep Kit (Zymo research; D6007), and molecular identification was achieved by internal transcribed spacer (ITS) region. Gene JET PCR Purification Kit (Thermo K0701) was used for purification of PCR product according to the manufacturer’s protocol. The resulting PCR products were sequenced by sequencing ready reaction kit (Applied Biosystems, Foster, CA, USA). Similar sequences via BLAST search database in the NCBI were compared with product sequence. Evolutionary study was directed in molecular evolutionary genetics analysis MEGA-x software [29–31].

### Extraction of Bioactive Secondary Metabolites

The bioactive secondary metabolites were extracted following the protocol suggested by Kjer et al. [22].Concretely, 2–3 mycelial plugs were removed from the actively growing edge of the pure fungal colony and inoculated in 1000 mL liquid Wickerham’s medium (0.3% yeast extract, 0.3% malt extract, 0.5% peptone, 1% glucose). Cultures were incubated at 28 °C in static and dark conditions for 21 days. After incubation period, the fermented broth was filtered through filter paper, and the metabolites produced by the fungus were extracted by equal volume of ethyl acetate and hexane separately. An equal volume of ethyl acetate and hexane separately was added to the filtrate and vigorously shaken for 5 min. The mixtures were transferred to separating funnels, and the organic layers of ethyl acetate and hexane were allowed to separate from the aqueous layers. Then, the ethyl acetate and hexane layers were allowed to dry at room temperature. The dried extracts were stored at 4 °C for further use.

### Phytochemical Screening of EA Extract

Qualitative screening of many phytochemicals (alkaloids, tannins, flavonoids, saponins, glycosides, phenols, steroids, and terpenoids) was evaluated according to Sarkar et al. [32], Kumar et al. [33], Onwukaeme et al. [34], Auwal et al. [35], and Raaman [36].

### Gas Chromatography-Mass Spectrometry (GC–MS) Analysis

EA crude extract of *A. alternaria* was injected to GC–MS to identify the metabolic compounds. GC–MS analysis was achieved using Agilent Technologies GC–MS 5977A operating at 70 eV and computer mass spectral library (NIST, 2011 version). The spectrum of the unknown constituents was matching with the available data stored in GC–MS libraries.

### In Vitro* Assessment of Antimicrobial Activity of Alternaria alternata Extracts*

The antimicrobial efficacy of *A. alternate* crude extracts was assessed against different human pathogenic microorganisms as against Gram-negative bacteria (*E. coli*,* P. vulgaris*, *P. aeruginosa*, and* K. pneumonia*), Gram-positive bacteria (*B. subtilis*, *S. aureus*, and *S. epidermidis*), and unicellular fungi (*C. albicans*). The methodology was performed using agar well diffusion assay as described by Gauchan et al. [37]. The dried extracts of the fungal strain were dissolved in dimethyl sulphoxide (DMSO). The microbial cultures were spread over the surface of sterilized nutrient agar and yeast extract peptone dextrose agar plates using sterile cotton swab. Wells of 6-mm diameter was made on the plates using a sterile borer. One hundred microliters of 1 mg/mL of fungal extracts dissolved in DMSO was added to the well. DMSO was used as negative control, and gentamycin (10 µg/mL) and fluconazole (20 mg/mL) were used as positive control. The plates were incubated at 37 °C for 24 h. The zone of inhibition was observed and measured. The inhibition zones with a diameter of less than 12 mm were considered having no antibacterial activity [38].

### Transmission Electron Microscopy (TEM)

In order to study the effect of EA crude extract of *A. alternata* on ultrastructure of the most sensitive bacteria, bacterial cells were collected by centrifugation at 4000 rpm for 10 min from 24-h old cultures grown on nutrient broth media and washed with distilled water; the samples were fixed in 3% glutaraldehyde, rinsed in phosphate buffer, and post-fixed in potassium permanganate solution for 5 min at room temperature. The samples were dehydrated in an ethanol series ranging from 10 to 90% for 15 min in each alcohol dilution and finally with absolute ethanol for 30 min. Samples were infiltrated with epoxy resin and acetone through a graded series until finally in pure resin. Ultrathin sections were collected on copper grids. Sections were then double stained in uranyl acetate followed by lead citrate. Stained sections were observed with a JEOL—JEM 1010 TEM at 80 kV at RCMB, Al-Azhar University [39, 40].

### Antioxidant Activity

Antioxidant activity of EA crude extract of *A. alternata* at various concentrations was carried out using DPPHH (2, 2-diphenyl-1Ppicrylhydrazyl) method by Khalil et al. [6] with minor modifications. Different concentrations of crude extracts (2000, 1000, 500, 250, 125, 62.5, 31.25, 15.62, and 7.81 µg/mL) were used to determine the scavenging of DPPH radicals. Antioxidant activity of standard and extracts was determined as DPPH scavenging activity (%): [((control absorbance – extract absorbance) / (control absorbance)) × 100] [41].

### In Vitro* Cytotoxicity Assay*

The cytotoxicity of EA crude extract of *A. alternata* at concentrations 4000—250 µg/mL was determined using the MTT protocol [42] with minor modification against normal Vero cell lines which collected from ATCC. The viability and inhibition percentages were calculated as shown in Eqs. 1 and 2 as follows:

1 $$\mathrm V\mathrm i\mathrm a\mathrm b\mathrm i\mathrm l\mathrm i\mathrm t\mathrm y\%=\frac{\mathrm{TestOD}}{\mathrm{Control}\;\mathrm{OD}}\mathrm X100$$

2 $$\mathrm{Inhibition \%}=100-\mathrm{Viability \%}$$

### Statistical Analysis

The data were expressed as the mean ± SDEV value, which was calculated by using Minitab 18 software extended with a statistical package and Microsoft Excel 365.

## Results and Discussion

### Isolation and Identification of Endophytic Fungus

**Fig. 1:**
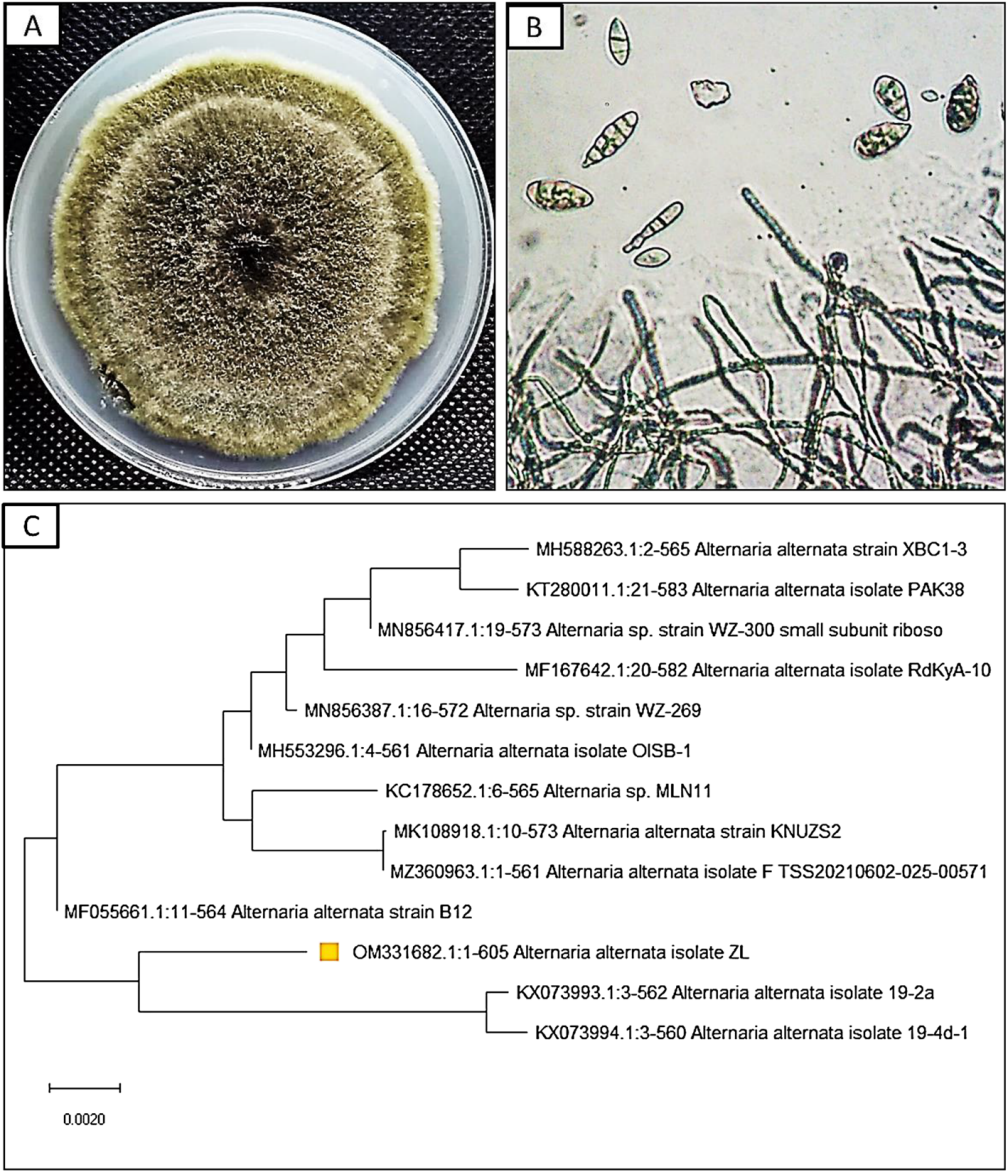
Morphological and molecular identification of *A. alternata* (**A**–**C**): **A** surface of culture on PDA; **B** conidiophore and conidia 400 × ; **C** phylogenetic tree

In this study, leaves of *Z. spina-christi* were used for isolation of fungal endophytes. During sterilization process, there was no mycelium growth on the control plates, indicating the success of sterilization procedure [43]. One fungal isolate was isolated from *Z. spina-christi* leaves; this strain was completely defined through traditional and molecular identification. Morphological identification revealed that diameter was 75–80 mm after 7 days, color is umber to olivaceous on PDA, and conidia comprising 1–7 transverse septa were narrow-ellipsoid or ovoid as shown in Fig. 1 A and B. The present fungus was morphologically similar to *Alternaria alternata.* Molecular identification confirmed that this strain is similar to *A. alternata* with 99% and recorded in gene bank with accession number OM331682. The phylogenetic analysis of fungal strains revealed 98% identity with ITS sequences of rRNA genes of related species using BLAST programs. Ascomycota and Deuteromycota are common for living fungal endophytes [44, 45]. Fungal endophytes *Alternaria* spp. were isolated in recent studies such as *A. alternata* [46–48] and *A. tenuissima* [6, 49].

### Phytochemical Analyses

Fungal endophytes are well known to produce large amount of novel antimicrobial and antioxidant compounds [50]. In the current study, EA extract contains on alkaloids, tannins, flavonoids, glycosides, phenols, and terpenoids as shown in Table 1. Flavonoids often inhibit fungal growth with various underlying mechanisms, including plasma membrane disruption, the induction of mitochondrial dysfunction, and inhibition of the following: cell wall formation, cell division, RNA and protein synthesis, and the efflux-mediated pumping system [51]. Glycosides serve as antifungal agent through acts as a specific inhibitor of glucan synthesis in cells and in vitro and lead to morphological changes in yeasts and molds [52]. Additionally, the antimicrobial activity may be referred to the presence of tannins because of its ability in molecular inhibitions of the cell membrane of microorganisms, where it decreases the development of complexes that keep its integrity, producing distortions and increasing their penetrability. Also, it affects extracellular microbial enzymes that lead to decrease in the essential compounds for cell development [53]. Phenolic compounds are a group of secondary metabolites holding functional hydroxyl group (-OH) linkage to aromatic hydrocarbon ring which are imperative bioactive compounds because their hydroxyl groups confer scavenging ability [54]. Terpenoids possess antitumor, anti-inflammatory, antibacterial, antiviral, and antimalarial effects, promote transdermal absorption, prevent and treat cardiovascular diseases, and have hypoglycemic activities [55].

**Table 1:** Phytochemical screening of EA crude extract of *A. alternata*

Phytochemical	EA extract
Alkaloids	** + **
Tannins	** + **
Flavonoids	** + **
Saponins	** − **
Glycosides	** + **
Phenols	** + **
Steroids	** − **
Terpenoids	** + **

### GC–MS Analysis of Bioactive Compounds

**Fig. 2:**
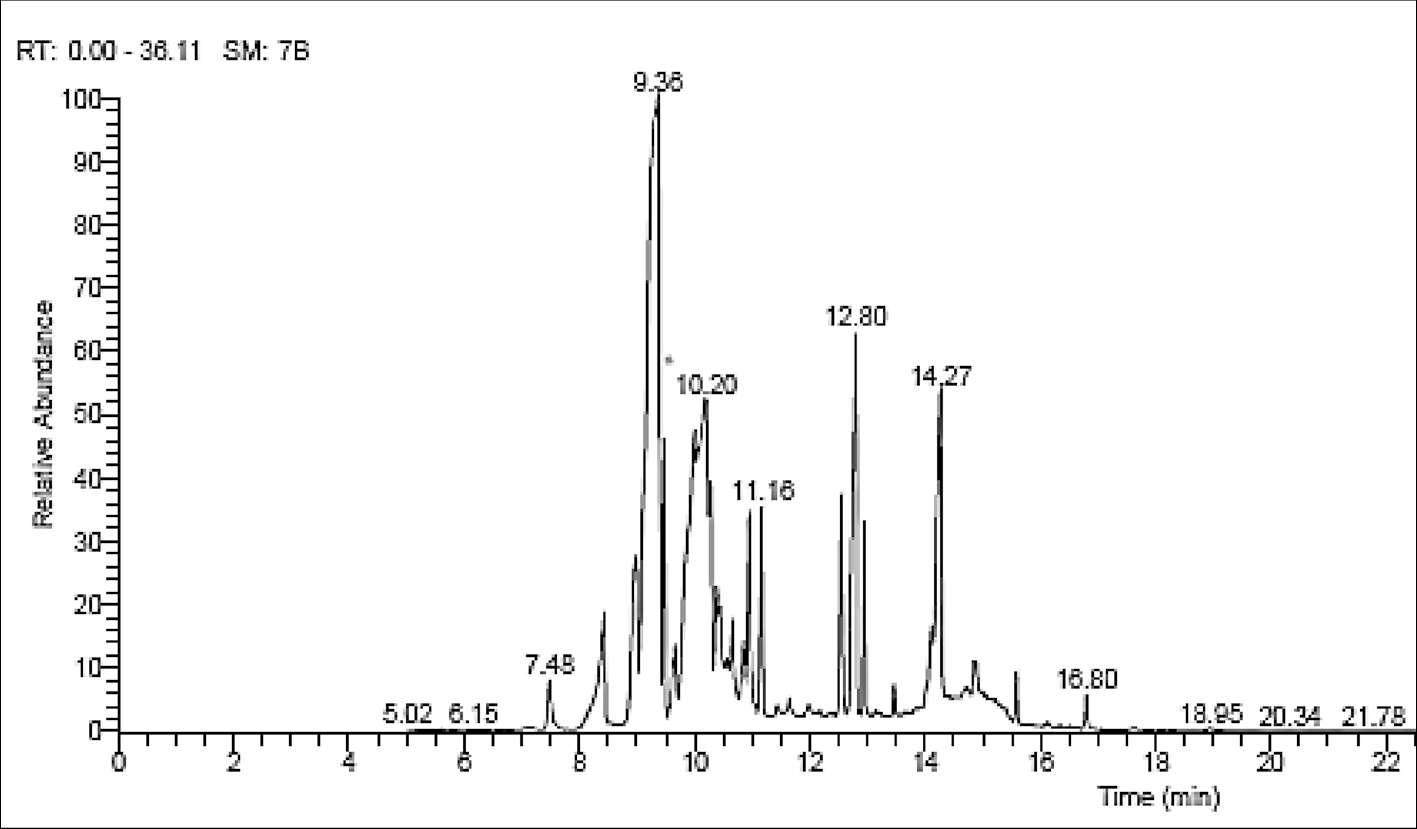
GC–MS chromatogram of EA crude extract of *A. alternata*

Gas chromatograph attached with mass spectrometer is one of the most accepted methods that is used for analyzing phytochemical compounds of natural origin because of their stability, sensitivity, and high efficiency [56]. Endophytic fungi living in medicinal plants can make the same pharmacological bioactive secondary metabolites in the same way as their host medicinal plants, which have been used for a long time in traditional medicine and even now are utilized for their health advantages [57, 58]. Results of GC–MS analysis of EA extract of *A. alternata* are illustrated in Table 2. Results revealed that EA extract of *A. alternata* contains 16 different compounds, where major compounds were oleic acid methyl ester and linolelaidic acid methyl ester with ratios 41.55 and 13.92%, respectively. Oleic acid is used as anti-inflammatory, anti-cancer, allergenic and insecticide properties, antioxidant, antimicrobial activities, cancer enzyme inhibitors [59, 60] [61]. On the other hand, minor compounds were hexadecanoic acid methyl ester, stearic acid methyl ester, cis-5,8,11,14,17-eicosapentaenoic acid, 9,12-octadecadienoic acid (Z,Z), 6,9,12-octadecatrienoic acid, methyl ester, cis-13-eicosenoic acid, methyl ester, eicosanoic acid, methyl ester, erucic acid, behenic acid, methyl ester, 1,2-benzenedicarboxylic acid (diisooctyl ester), tetracosanoic acid, methyl ester, linoleic acid ethyl ester, hexacosanoic acid, methyl ester, and stigmastan-3,5-diene with ratios 3.11, 5.81, 2.13, 0.95, 1.09, 3.20, 2.99, 3.48, 8.85, 2.17, 7.43, 0.36, 0.52, and 0.46%, respectively. These compounds have different biological activities such as antimicrobial, antioxidant, hypocholesterolemic, nematicide, pesticide, antiandrogenic, anticolorectal cancer activity, hepatoprotective, antihistamine, hypocholesterolemic, anti-eczemic, antistaphylococcal, antihypertensive, and antiulcer activities as illustrated in Table 2 and Fig. 2.

**Table 2:** GC–MS analysis of EA crude extract of *A. alternata*

	Compound	Rt (min)	Peak area %	Activity	References
1	Hexadecanoic acid methyl ester	8.43	3.11	Antioxidant, hypocholesterolemic, nematicide, pesticide, antiandrogenic	[62]
2	Oleic acid methyl ester	9.36	41.55	Anti-inflammatory, anticancer, allergenic, and insecticide properties, antioxidant, antimicrobial activities, cancer enzyme inhibitors	[59, 60] [61]
3	Stearic acid methyl ester	9.47	5.81	Antibacterial	[63]
4	Linolelaidic acid methyl ester	9.99	13.92	No activity reported	-
5	cis-5,8,11,14,17-Eicosapentaenoic acid	10.39	2.13	Antibacterial, anticolorectal cancer activity	[64] [65]
6	9,12-Octadecadienoic acid (Z,Z)	10.65	0.95	Hepatoprotective, antihistamine, hypocholesterolemic, anti-eczemic activity	[66]
7	6,9,12-Octadecatrienoic acid, methyl ester	10.84	1.09	No activity reported	-
8	cis-13-Eicosenoic acid, methyl ester	10.95	3.20	No activity reported	-
9	Eicosanoic acid, methyl ester	11.16	2.99	No activity reported	-
10	Erucic acid	12.55	3.48	Antibacterial activity	[67]
11	Behenic acid, methyl ester	12.80	8.85	No activity reported	-
12	1,2-Benzenedicarboxylic acid (diisooctyl ester)	12.94	2.17	Antimicrobial, fungitoxic, and cytotoxic activity, antioxidant	[68] [69] [70]
13	Tetracosanoic acid, methyl ester	14.26	7.43	No activity reported	-
14	Linoleic acid ethyl ester	14.85	0.36	Antibacterial	[61]
15	Hexacosanoic acid, methyl ester	15.58	0.52	No activity reported	-
16	Stigmastan-3,5-diene	16.80	0.46	Antistaphylococcal, antihypertensive and antiulcer activities	[71]

### Antimicrobial Activity

**Fig. 3:**
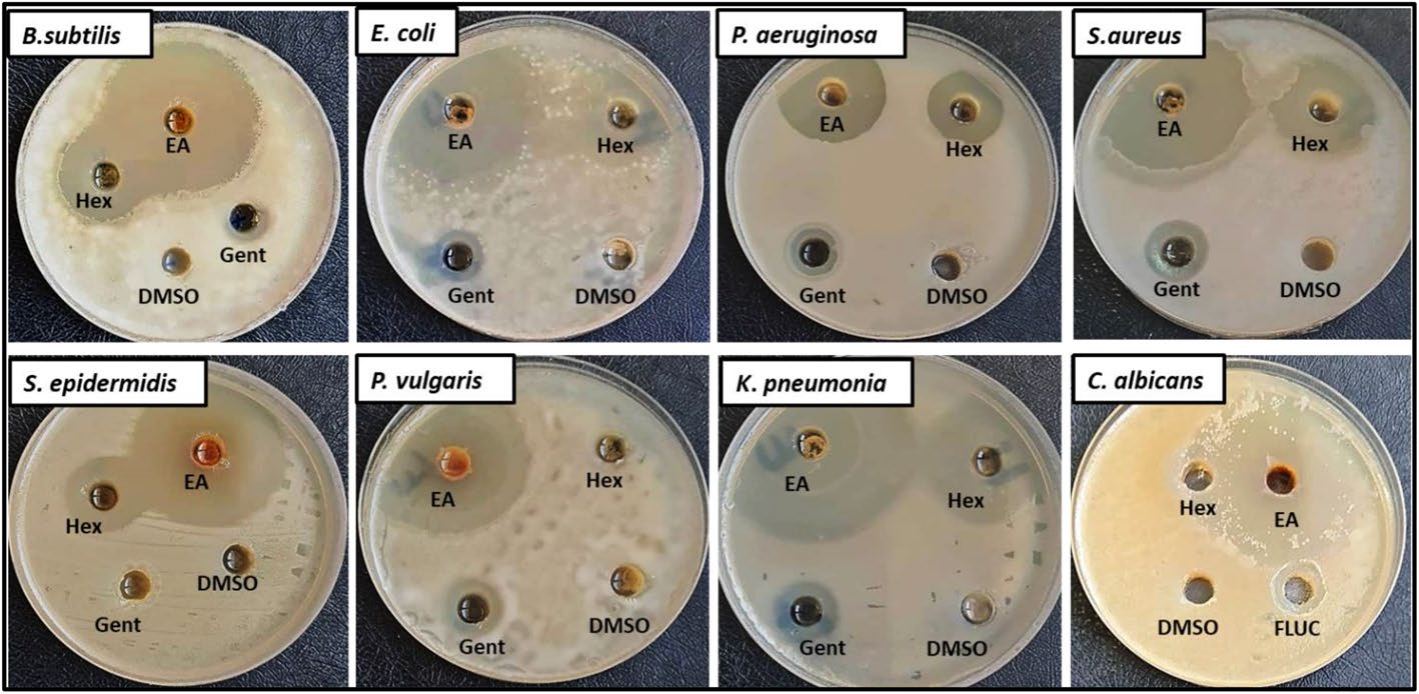
Antimicrobial activity of *Alternaria alternata* crude extracts against human pathogenic microorganisms by agar well diffusion method

The EA and hexane crude extracts obtained from *A. alternata* were evaluated for their antimicrobial activity against human pathogenic microorganisms by agar well diffusion method. Data presented in Table 3 clearly indicated that both crude extracts exhibited different degree of inhibition as compared to controls. Interestingly, ethyl acetate displayed a strong inhibitory activity against all Gram-negative and Gram-positive bacteria and unicellular fungi, whereas the hexane crude extract exhibited weak growth inhibition against tested organisms and did not affect the growth of *P. vulgaris*. The maximum inhibitory activity of the EA crude extract was recorded against *K. pneumonia* with inhibition zone of 49 ± 0.05 mm followed by *S. epidermidis* 43 ± 0.00 mm, *E. coli* 42 ± 0.2 mm, *P. vulgaris* 41 ± 0.08 mm, and *B. subtilis* 40.06 ± 0.1 mm as shown in Fig. 3*.* These results demonstrated that EA crude extract presented high broad-spectrum activity as compared to hexane extract. That means ethyl acetate contains the maximum number/concentration of bioactive compounds which directly or indirectly influences the inhibition zone. Activity of secondary metabolites is attributed to tier ability to cell wall synthesis and depolarizes the cell membrane, inhibition of protein synthesis, inhibition of nucleic acid synthesis, and metabolic pathways inhibition in bacteria [72, 73]. Previous reports evaluated that the activity of the prepared extracts from the leaves and fruits depends on the active ingredients as well as the polarity of the ingredients [74, 75]. In addition, several studies showed that the extracts of different polarity give potentially different pharmacological and toxicological activities [76, 77]. Our results are in accordance to Al Mousa et al. [78] who stated that the EA crude extract obtained from *Alternaria tenuissima* AUMC14342 gave the highest antimicrobial activity against *P. aeruginosa*, *S. aureus*, *Fusarium solani*, and *Aspergillus niger* at concentration 30 mg/mL using disc diffusion assay. Moreover, our results are in agreement with Techaoei et al. [79] who found that the EA crude extract of *A. alternata* isolated from lotus displayed more potential against both *S. epidermidis* and *Methicillin-resistant Staphylococcus aureus* (MRSA). Similarly, Tang et al. [80] reported that the EA crude extract of *Penicillium oxalicum* had antibacterial effects against all the tested bacteria with MIC between 0.50 and 2 mg/mL. Also, only the EA extract of *Simplicillium* sp. showed antibacterial effect against *E. coli*, *P. aeruginosa*, *B. subtilis*, and *S. aureus* with MIC of 0.5, 1, 2, and 1 mg/mL, respectively. Chatterjee et al. [81] revealed that the EA extract of endophytic fungus *A. alternata* VN3 isolated from *Vitex negundo* L. was also effective against both Gram-positive and Gram-negative bacteria.

**Table 3:** Effect of EA and hexane crude extracts of *A. alternata* on growth inhibition of pathogenic microorganisms

Microbial strains	Fungal crude extracts Diameter inhibition zones (mm) against pathogenic microorganisms			
	DMSO (control)	EA (1 mg/mL)	Hexane (1 mg/mL)	Gentamycin (10 µg/mL)/fluconazole (20 mg/mL)
***E. coli***	0	42 ± 0.2	20 ± 0.1	14 ± 0.2
***P. aeruginosa***	0	28 ± 0.005	20.3 ± 0.01	16 ± 0.01
***K. pneumonia***	0	49 ± 0.05	21 ± 0.3	15 ± 0.2
***P. vulgaris***	0	41 ± 0.08	0.00	12.3 ± 0.01
***S. aureus***	0	39 ± 0. 1	28.3 ± 0.1	13.6 ± 0.02
***S. epidermidis***	0	43 ± 0.00	20 ± 0.00	9 ± 0.00
***B. subtilis***	0	40.06 ± 0.1	24.6 ± 0.05	6.3 ± 0.05
***C. albicans***	0	33 ± 0.11	11 ± 0.17	9 ± 0.00

*EA* ethyl acetate, ± SD

### Ultrastructure Study

**Fig. 4:**
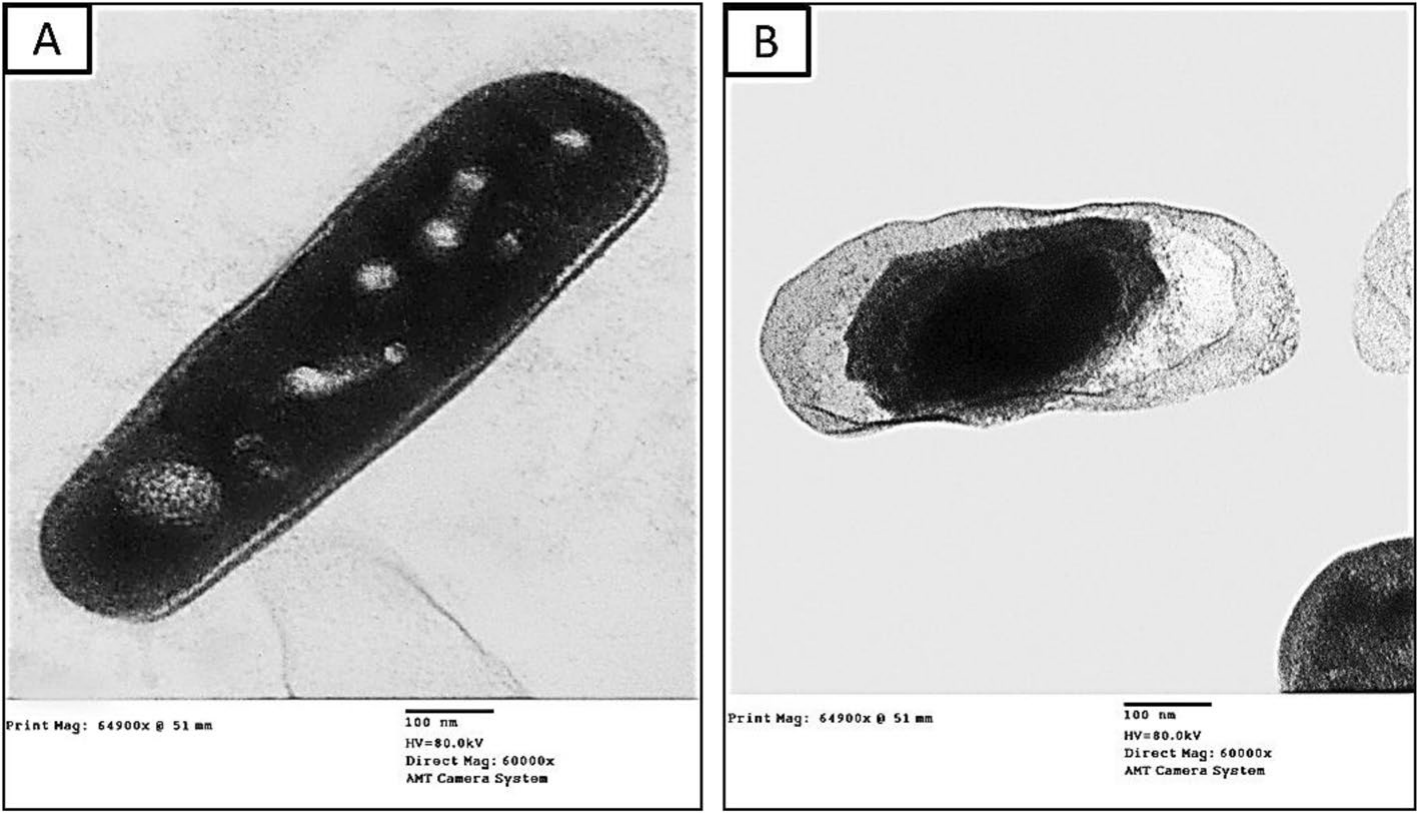
Transmission electronic micrographs of *K. pneumonia*. **A** Control (untreated) and **B** treated cell with EA crude extract of *A. alternata*. Scale Bar = 100 nm

To confirm antibacterial activity of EA extract *A. alternata*, ultrastructure of treated *K. pneumonia* by this extract was carried out as shown in Fig. 4. Rod-shaped cells, smooth continuous cell wall and cell membrane, homogeneous electron dense cytoplasm, and normal electron lucent zone between cell wall and cell membrane are seen in this transmission electron micrograph of typical *K. pneumonia* (Fig. 4A). On the other hand, treated *K. pneumonia* with EA crude extract of *A. alternata* displayed deformed cells with rough uneven cell walls appear to be seeping from the damaged membrane with electron lucent patches emerge in the cytoplasm; remarkably elucidated destruction of the cell wall and cell membrane. These treated cells obviously revealed leakage of cytoplasmic materials, concerned in the center of the cell leave large space in between as shown in Fig. 4B. Antimicrobial agents affect bacterial cell membrane leading to complete damage of the cells [82]. Nath and Joshi [83] studied the effect of ethanolic extract of endophytic fungus *Glomerella acutata* EF15 on *K. pneumonia* and found that bacterial cells appeared crumpled and shrunken, and cavity formation was prominent on the cell membrane of the bacteria which lead to complete damage.

### Antioxidant Activity

**Fig. 5:**
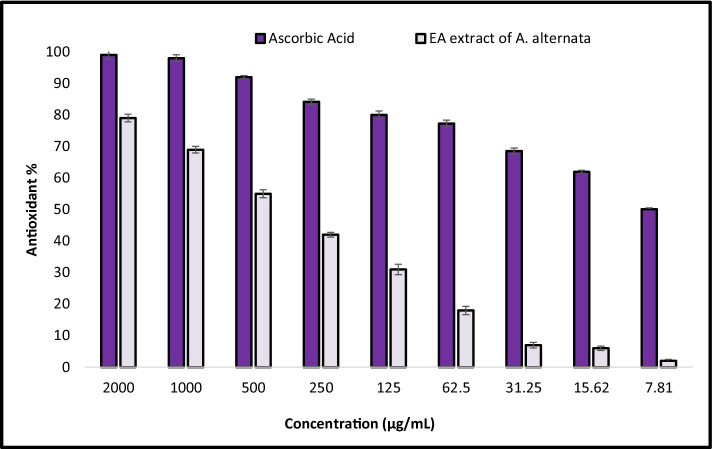
Antioxidant activity of EA crude extract of *A. alternata*

This work was designed to obtain fungal endophytes with promising antioxidant activities from *Z. spina-christi* plants. Biological reactions usually produce reactive oxygen species (ROS) as by-products which causes cell death due to oxidative damage to biological materials [84]. Beating the harmful effect of ROS in human organs, external source of antioxidant should be useful. However, one of the main properties of antioxidant molecules is their capability to hold and balance free radicals [85]. Antioxidants have been considered therapy agents where they possess anti-atherosclerotic, anti-inflammatory, antitumor, anticarcinogenic, antimutagenic, and antimicrobial properties. Antioxidants are frequently found naturally in medicinal herbs, vegetables, and fruits. In our study, EA crude extract of *A. alternata* was assessed as antioxidant using DPPH method as shown in Fig. 5. Results revealed that EA crude extract of *A. alternata* exhibited promising antioxidant activity as compared to ascorbic acid, where activity at 500–2000 µg/mL was above 50%. Also, results illustrated that IC_50_ of EA crude extract of *A. alternata* was 409 µg/mL. This activity is attributed to presence phenolic compounds which are confirmed by phytochemical screening and GC–MS. Ibrahim et al. [86] reported that EA crude extract of *Alternaria* sp. showed potential antioxidant activity and IC_50_ was 520 µg/mL. Moreover, Khiralla et al. [87] isolated *Alternaria* sp. from leaves of *Calotropis procera*, where it exhibited antioxidant activity with MIC 236 µg/mL. Another study confirmed that EA extract of *Alternaria* sp. (ML4) had DPPH scavenging activity of 85.20% at the concentration of 300 µg/mL and high reducing power activity [88].

### Cytotoxic Activity

**Fig. 6:**
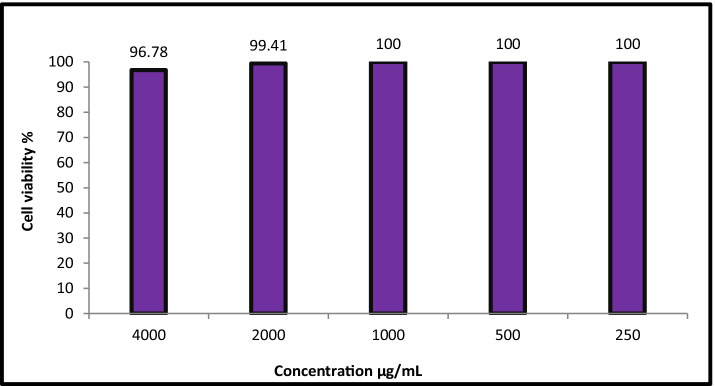
Cytotoxicity of EA crude extract of *A. alternata* against Vero cell line

Evaluation of cytotoxicity of the natural metabolic products is the first step to determine their safety on noncancerous human cells [89]. Cytotoxicity of EA crude extract of *A. alternata* was determined toward Vero normal cell line as illustrated in Fig. 6. Results revealed that concentration of EA crude extract of *A. alternata* at <  = 1000 µg/ml did not show any toxicity on Vero cell line. Also, IC_50_ was greater than 4000 µg/mL, where if the IC_50_ is ≥ 90 µg/mL, the compound is classified as not cytotoxic [90]. Eventually, EA crude extract of *A. alternata* is non-toxic and safe for use.

## Conclusion

In the current study, promising endophytic *A. alternata* was isolated from leaves of *Z. spina-christi* and deposited in gene bank with accession number OM331682. Bioactive compounds which were produced by endophytic *A. alternata* were analyzed and determined through GC–MS and phytochemical analyses. Crude extract of *A. alternata* has promising antibacterial and antifungal properties against Gram-negative, Gram-positive, and unicellular fungi. Moreover, ultrastructure study confirmed damaging cell wall and cell membrane and leakage of cytoplasmic materials. Furthermore, this extract has potential antioxidant activity as well as no toxicity on normal cell line. Eventually, the crude extract of endophytic *A. alternata* is recommended as bioactive compounds for different biological applications.
